# Genetic map construction and QTL analysis of leaf-related traits in soybean under monoculture and relay intercropping

**DOI:** 10.1038/s41598-019-39110-8

**Published:** 2019-02-25

**Authors:** Dai-Ling Liu, Si-Wei Chen, Xin-Chun Liu, Feng Yang, Wei-Guo Liu, Yue-Hui She, Jun-Bo Du, Chun-Yan Liu, Wen-Yu Yang, Xiao-Ling Wu

**Affiliations:** 1 Sichuan Engineering Research Center for Crop Strip Intercropping System, Sichuan Agriculture University, Chengdu, 611130 P. R. China; 2 College of Agronomy, Sichuan Agriculture University, Chengdu, 611130 P. R. China; 3 Industrial Crop Research Institute, Sichuan Academy of Agricultural Sciences, Chengdu, 610300 P. R. China

## Abstract

Soybean (*Glycine max* L.) is an important food and oil crop widely planted by intercropping in southwest China. The shade caused by intercropping changes plant growth traits, such as soybean leaf and dry mass, thereby reducing yields. To improve the yield and elucidate the genetic mechanism of the leaf-related traits in intercropped soybeans, we measured the F_6:7–8_ recombinant inbred lines (RILs) derived from the cross of ‘Nandou 12’ and ‘Jiuyuehuang’ for six leaf-related traits under monoculture and relay intercropping in 2015 and 2016. We found 6366 single-nucleotide polymorphisms (SNPs) markers that covered the whole genome of soybean distributed in 20 linkage groups, which spanned 2818.67 cM with an average interval of 0.44 cM between adjacent markers. Nineteen quantitative trait loci (QTLs) were detected in two environments in 2 years. Three candidate genes associated to leaf-related traits were found according to gene expression and GO enrichment analyses. These results revealed the susceptibility of leaf phenotype to shading and helped elucidate the mechanisms that control leaf-related traits.

## Introduction

Soybean (*Glycine max* L.) is an important crop with a high protein and oil content, and is widely used for food and feed and chemical industry. In China, with the increasing demand for soybean and the shrinking of planting area, abundant resources and diversified planting systems should be maximized to develop the soybean industry and to breed high-yield varieties.

Maize-soybean relay strip intercropping remarkably increases the planting area and production of soybean and maintains the yield of maize^1,2^. In this planting pattern, soybean is subjected to shading at the co-growth stage. The morphological and photosynthetic characteristics of soybean leaves are considerably affected under shading. In shading, soybean leaf size inhibited by controlling cell proliferation and enlargement^3^, and the dry mass distribution increases in stem but decreases in leaves^4^. Poor light also reduces the specific leaflet weight and the photosynthetic rate^5^. In serious cases, photosynthetic capacity and yield decrease^1^.

More than 88 quantitative trait loci (QTLs) associated with soybean leaf-related traits have been reported. Of these QTLs, 64 QTLs for leaf-related traits in Soybase (https://soybase.org), including 35 QTLs for the leaflet area^6–12^, 2 QTLs for specific leaflet area^11^, 1 QTL for leaflet weight^11^, 15 QTLs for specific leaflet weight^8,12^, and 11 QTLs for leaflet ash^13^. Other scholars also detected some QTLs related leaf traits of soybean. Shi *et al*.^14^ reported 8 QTLs for leaf length and 9 QTLs for leaf width by using recombination inbred lines (RILs) derived from a cross between ‘Charleston’ and ‘Dongnong 594’. Kim *et al*.^15^ identified 7 QTLs for leaflet length and width in two F_2:10_ populations. The QTLs of leaf traits associated with shading were relatively few. Only few QTLs of other crops have been identified under poor light. For example, 5 QTLs are related to the increase in leaf area in cucumber^16^, and 6 QTLs are associated with the specific leaf weight in maize^17^. However, the QTL of soybean in shading has yet to be reported.

In the present study, we analyzed the leaf phenotype under monoculture (M) and relay intercropping (RI), developed a high-density soybean molecular genetic map by using a specific-locus amplified fragment sequencing (SLAF-seq) technology, and identified the QTL for leaf-related traits by utilizing RILs derived from ‘Nandou 12’ and ‘Jiuyuehuang’, and determined the main effect of QTLs and select candidate genes that may influence the leaf phenotype through Gene Ontology (GO) enrichment analysis.

## Results

### Phenotypic variance and correlation

**Figure 1:**
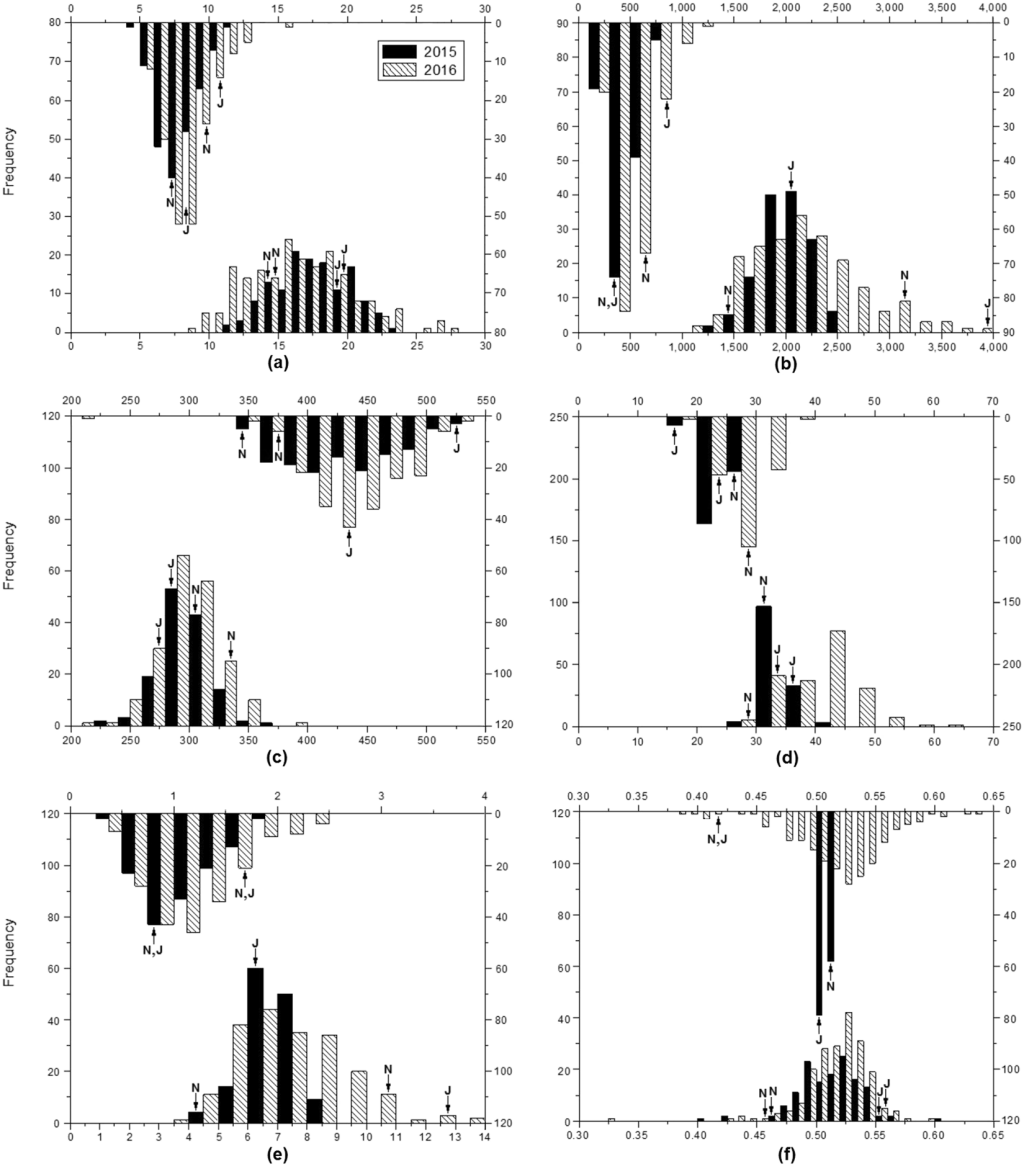
Histograms of frequency distribution for compound leaf number (**a**), total leaflet area (**b**), specific leaflet area (**c**), specific leaflet weight (**d**), leaflet dry weight (**e**) and leaflet dry weight ratio (**f**) in the RILs population. In each panel, the upward side is the distribution of the phenotypic data in relay intercropping (RI), whereas the downward side is in the monoculture (M). The black bars represent the data in 2015, and the slash bars correspond to the data in 2016. Arrows indicate the phenotypic value ranges of ‘Nandou 12’ (N) and ‘Jiuyuehuang’ (J) in 2 years.

‘Jiuyuehuang’ had greater compound leaf number (CLN), total leaflet area (TLA), and leaflet dry weight (LDW) in monoculture and relay intercropping than ‘Nandou 12’ (Table 1). ‘Nandou 12’ had greater specific leaflet weight (SLW) and leaflet dry weight ratio (LDWR) in relay intercropping than ‘Jiuyuehuang’, but opposite in monoculture, and specific leaflet area (SLA) of ‘Jiuyuehuang’ was similar. The phenotypic distribution and the normality of the RILs were shown in Fig. 1. The results of variance analysis indicated the significant variation in CLN, TLA, SLA, SLW, and LDW except LDWR between monoculture and relay intercropping. CLN, TLA, SLW, and LDW in relay intercropping were significantly lower than those in monoculture (*P* < 0.05). By contrast, SLA in relay intercropping was significantly higher than that in monoculture. These results revealed the susceptibility of leaf phenotype to shading.

**Table 1:** Phenotypic performance of leaf-related traits in parents and RILs under monoculture (M) and relay intercropping (RI) in 2015 and 2016.

Traits	Test	Year	Parents		RILs				
			Nandou 12	Jiuyuehuang	Mean	Range	SD	CV%	P (S-W)
CLN	M	2015	13.34 ± 0.47B	19.16 ± 0.23A	17.54	11.67	2.63	14.99	0.26
		2016	14.66 ± 1.31b	19.92 ± 2.83a	16.38	21.33	3.88	23.69	0.02
	RI	2015	7.67 ± 0.47a	8.00 ± 0.47a	7.63	6.50	1.34	17.56	0.40
		2016	9.67 ± 0.32a	10.75 ± 1.62a	8.15	7.33	1.57	19.26	0.00
TLA (cm^2^)	M	2015	1305.00 ± 58.65B	2025.00 ± 5.54A	2013.13	1216.91	247.43	12.29	0.16
		2016	3201.00 ± 60.20B	4131.00 ± 273.00A	2162.52	2751.04	527.94	24.41	0.00
	RI	2015	309.90 ± 2.40B	453.50 ± 4.23A	453.22	647.30	132.83	29.31	0.06
		2016	563.80 ± 26.44B	778.40 ± 17.18A	503.14	970.12	178.33	35.44	0.02
SLA (cm^2^/g)	M	2015	311.20 ± 0.92 A	280.70 ± 0.25B	297.39	125.24	20.10	6.76	0.16
		2016	336.80 ± 11.82 A	273.70 ± 8.56B	297.69	178.77	24.85	8.35	0.16
	RI	2015	331.50 ± 1.41B	554.80 ± 4.87A	434.32	192.71	46.20	10.64	0.09
		2016	373.40 ± 4.37B	446.10 ± 16.02A	438.53	186.74	36.64	8.36	0.27
SLW (g/m^2^)	M	2015	32.95 ± 1.25a	35.61 ± 0.00a	33.78	12.38	2.11	6.25	0.25
		2016	29.80 ± 1.03a	33.95 ± 2.22a	40.59	36.50	5.93	14.61	0.00
	RI	2015	29.04 ± 1.47a	18.36 ± 0.31b	23.81	10.39	2.48	10.42	0.04
		2016	25.65 ± 1.08a	22.58 ± 1.59a	27.48	18.44	3.57	12.99	0.13
LDW (g)	M	2015	4.68 ± 0.16a	6.17 ± 0.48a	6.80	4.31	0.82	12.06	0.08
		2016	10.24 ± 0.19B	12.78 ± 0.49A	7.37	9.57	1.85	25.10	0.00
	RI	2015	0.78 ± 0.21a	0.81 ± 0.02a	1.04	1.52	0.30	28.85	0.00
		2016	1.54 ± 0.08a	1.70 ± 0.13a	1.17	2.07	0.44	37.61	0.00
LDWR	M	2015	0.46 ± 0.02b	0.56 ± 0.03a	0.51	0.21	0.02	0.04	0.00
		2016	0.46 ± 0.03B	0.56 ± 0.01A	0.51	0.27	0.03	0.05	0.00
	RI	2015	0.52 ± 0.07a	0.50 ± 0.01a	0.51	0.01	0.00	0.01	0.00
		2016	0.42 ± 0.02a	0.42 ± 0.04a	0.51	0.25	0.04	0.08	0.00

In parents, data are means ± SD (n = 3). Statistical significance assessed by Duncan’s t-test. The capital letters and the small letters on the right side of data in parents denote significance level of *P* < 0.01 and 0.05 in the same row, respectively.

Supplementary Table S1 shows the Pearson correlation coefficients. Significantly positive correlations were found between CLN and TLA, CLN and LDW, and TLA and LDW, whereas significantly negative correlations were observed in SLA and SLW in two different planting patterns in both years.

### Genetic map

**Figure 2:**
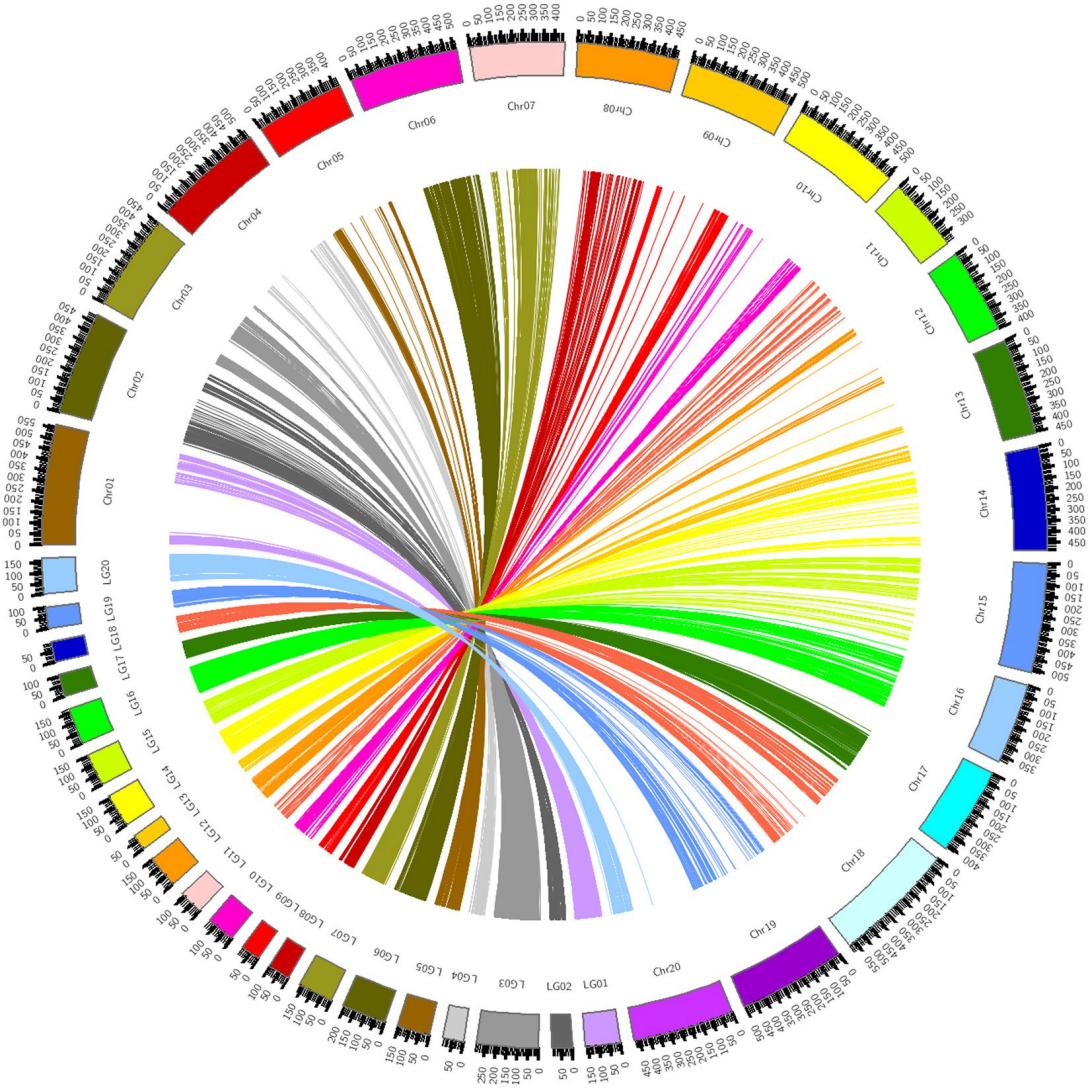
Collinearity of the genetic map and the physical map. The corresponding relationship and the position relationship between the soybean chromosomes (Chr) and the linkage group (LG) of the genetic map are shown.

The whole genome resequencing of the two parents and the SLAF-seq of RILs population revealed that 221.67 Gb sequence reads were obtained. The read number was 1,018.34 Mb, and the average sequencing Q30 was 94.70%. The average GC content was 38.69%, and the sample GC distribution was normal. A total of 931,337 high-quality polymorphic single-nucleotide polymorphism (SNP) sites were detected, and 6,552 SNPs were used for high-density linkage map construction. After filtering out the markers that will affect collinearity, there were 6,366 SNPs being mapped into 20 linkage groups by using Highmap. The total genetic distance was 2,818.67 cM, and the average interval ranged from 0.27 cM to 0.70 cM by chromosome with an overall average marker distance of 0.44 cM. In Table 2, Chr03 (290.11 cM) was the largest and had the highest number of markers (773), whereas Chr13 (76.88 cM) was the shortest. Chr04 had the fewest markers (94). Chr02 had the minimal average map distance between markers (0.27 cM). Approximately 99.10% of the intervals between adjacent markers on the genetic map were less than 5 cM, and only the intervals between adjacent markers on Chr01 were all less than 5 cM. The spearman correlations of 20 linkage groups between the linkage map and the genome map were also analyzed. Each correlation coefficient of 20 linkage groups was nearly close to 1, which showed a relatively high collinearity between the linkage groups and the soybean reference genome (Fig. 2). In the 20 linkage groups, Chr09 had the highest collinearity with a correlation coefficient (0.999).

**Table 2:** Characteristics of the 20 chromosomes in the genetic map with 6366 SNPs.

Linkage group ID	Marker number	Total distance (cM)	Average distance (cM)	Max gap (cM)	Gap < 5 cM (%)	Correlation coefficient
Chr01	343	165.45	0.48	4.90	100.00	0.960
Chr02	366	97.33	0.27	7.06	98.90	0.814
Chr03	773	290.11	0.38	6.03	99.61	0.800
Chr04	94	99.84	1.06	12.90	96.77	0.908
Chr05	314	155.91	0.50	10.02	99.36	0.936
Chr06	741	208.60	0.28	9.28	99.73	0.849
Chr07	299	165.90	0.55	16.29	99.66	0.948
Chr08	309	103.81	0.34	17.32	99.68	0.981
Chr09	247	93.69	0.38	7.86	99.19	0.999
Chr10	204	136.72	0.67	17.66	98.03	0.969
Chr11	182	116.70	0.64	15.48	97.24	0.938
Chr12	133	152.11	1.14	11.13	96.97	0.973
Chr13	157	76.88	0.49	11.67	98.08	0.998
Chr14	263	158.89	0.60	11.70	98.85	0.971
Chr15	219	152.59	0.70	8.85	97.71	0.929
Chr16	434	170.16	0.39	8.97	99.08	0.908
Chr17	363	106.74	0.29	7.75	99.72	0.802
Chr18	338	97.93	0.29	7.19	98.81	0.968
Chr19	277	100.05	0.36	10.51	98.91	0.998
Chr20	310	169.28	0.55	10.02	99.68	0.972
Total	6,366	2,818.67	0.44	17.66	99.10	—

The closer the Spearman correlation coefficient is to 1, the better the collinearity.

### QTL analysis

**Figure 3:**
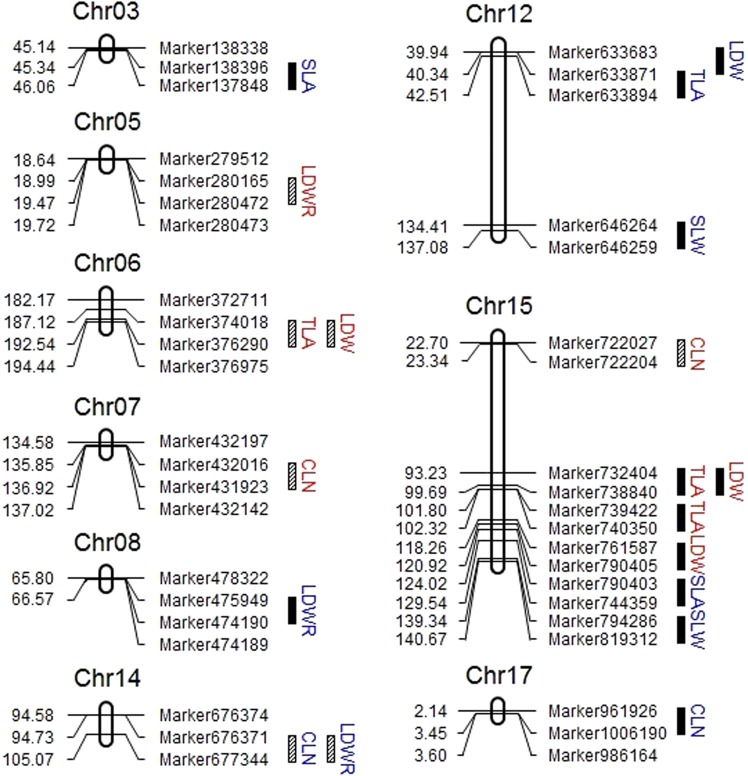
The QTL mapping of six leaf-related traits under monoculture and relay intercropping in 2015 and 2016. The linkage groups of QTLs are listed. The position of each marker is shown on the left side of the linkage group, while QTLs are marked on the right. The black cubes are the QTLs detected under monoculture, and the slash cubes are the QTLs detected under relay intercropping. The blue letters represent the QTLs in 2015, and the red letters represent the QTLs in 2016.

A total of 19 QTLs for six leaf-related traits were identified in nine different chromosomes (Chr03, Chr05, Chr06, Chr07, Chr08, Chr12, Chr14, Chr15, and Chr17) in 2 years (Fig. 3 and Table 3), and 12 QTLs were identified in monoculture with their phenotypic variance effect ranging from 4.00% (*qLDW15-119*) to 14.47% (*qLDW15-98*), and seven QTLs in relay intercropping accounted for 5.37% (*qCLN15-23*) to 13.88% (*qLDWR5-19*) of phenotypic variations. Furthermore, 4 QTLs associated with SLA and SLW were only detected in monoculture, whereas 15 QTLs related to CLN, LDW, and LDWR were detected in monoculture and relay intercropping. Of 19 leaf-related QTLs, sixteen QTLs showed positive additive effects from ‘Jiuyuehuang’, whereas three QTLs were negative additive effects with ‘Nandou 12’. In addition, 3 QTL hotspots flanked by Marker374018 and Marker376290 on Chr06, Marker676371 and Marker677344 on Chr14, and Marker732404 and Marker728840 on Chr15 were identified.

**Table 3:** QTLs detected in Nandou 12 × Jiuyuehuang RILs under monoculture and relay intercropping in 2015 and 2016.

Traits	Test	Year	QTL	Chr.	Position	Left Marker	Right Marker	LOD	Add	PVE (%)
CLN	M	2015	*qCLN17-3*	17	3	Marker961926	Marker1006190	6.72	0.81	10.71
	RI	2015	*qCLN14-100*	14	100	Marker676371	Marker677344	3.72	0.37	8.38
		2016	*qCLN7-136*	7	136	Marker432016	Marker431923	3.98	0.50	7.37
			*qCLN15-23*	15	23	Marker722027	Marker722204	2.94	−0.44	5.37
TLA	M	2015	*qTLA12-41*	12	41	Marker633871	Marker633894	2.78	0.01	10.69
		2016	*qTLA15-99*	15	99	Marker732404	Marker738840	23.74	0.04	14.06
			*qTLA15-102*	15	102	Marker739422	Marker740350	10.17	−0.03	5.04
	RI	2016	*qTLA6-192*	6	192	Marker374018	Marker376290	4.21	0.01	9.24
SLA	M	2015	*qSLA3-46*	3	46	Marker138396	Marker137848	2.70	0.00	5.39
			*qSLA15-129*	15	129	Marker790403	Marker744359	3.39	0.00	6.94
SLW	M	2015	*qSLW12-137*	12	137	Marker646264	Marker646259	2.70	0.00	4.92
			*qSLW15-140*	15	140	Marker794286	Marker819312	3.61	0.00	6.91
LDW	M	2015	*qLDW12-40*	12	40	Marker633683	Marker633871	4.40	0.26	11.00
		2016	*qLDW15-98*	15	98	Marker732404	Marker738840	11.87	1.04	14.47
			*qLDW15-119*	15	119	Marker761587	Marker790405	3.67	−0.53	4.00
	RI	2016	*qLDW6-192*	6	192	Marker374018	Marker376290	4.37	0.14	9.60
LDWR	M	2015	*qLDWR8-66*	8	66	Marker475949	Marker474190	4.37	0.01	9.73
	RI	2015	*qLDWR14-95*	14	95	Marker676371	Marker677344	2.66	0.00	5.95
		2016	*qLDWR5-19*	5	19	Marker280165	Marker280472	6.00	0.01	13.88

Add, additive effect; PVE, percentage of phenotypic variation explained.

The phenotypic data on leaf traits and genotypic data on markers were analyzed by QTL.gCIMapping.GUI software. The results indicated that QTL *qLDW15-98* detected in 2016 under monoculture was precisely determined at 96.00 cM on Chr15 with 14.59% of phenotypic variations, being in marker interval of Marker732404–Marker738840. In addition, there was a QTL (LOD = 3.51) associated to CLN in 2015 under monoculture closing to *qCLN*17*-*3, which was located on 9.36 cM between Marker969731–Marker969731 with 9.99% of phenotypic variations.

### GO enrichment analysis based on co-located QTLs

In terms of co-located QTL, 3 QTL hotspots were investigated in detail. Our results revealed that 6 QTLs associated with four traits explained 5.95% to 14.47% of the phenotypic variations. To further identify the genes that were related to the leaves in the three regions, we retrieved gene calls and annotations by using Glyma.Wm82.a2.v1 gene model from Soybase (https://soybase.org). Using GO enrichment analysis, we found 160, 66, and 235 genes within the three clusters on Chr06, Chr14, and Chr15, respectively. Among them, 129 annotated genes were closely related to leaves and could be classified into five groups (Table 4). The first group contained 45 genes associated with phytohormone regulation, including hormones, such as auxin, gibberellin, ethylene, and abscisic acid, which could be related to leaflet number. The second group included 48 genes related to photosynthesis, including those that played a role in the process of photosynthesis, light quality, light intensity, and relevant carbohydrates, which were implicated in leaflet dry weight and area^18^. The third group comprised 85 genes associated with cellular processes, including cell division, cell differentiation, and cell growth, which might affect the leaflet area. The fourth group consisted of 11 genes associated with morphogenesis, including photomorphogenesis, cell morphogenesis, and leaf morphogenesis, which could be related to leaflet shape and area. The fifth group was composed of 32 genes associated with leaf development and leaf growth-related factors, such as water, nitrogen, and phosphorus, which directly influenced leaves. In terms of the predicted function of the five groups, three predicted genes (*Glyma06G296500*, *Glyma14G087100*, *Glyma15G154000*) were selected as the best candidate genes that might affect the leaf phenotype because they referred to various biological processes (Supplementary Table S2). *Glyma06G296500* encodes a GH3-related gene involved in red light-specific hypocotyl elongation. GO analysis showed that this gene is also involved in response to auxin and light stimulus in *Arabidopsis thaliana*. *Glyma14G087100* encodes cyclin-dependent protein kinase 3 and participates in cell cycle, cell division, and cell cycle regulation. *Glyma15G154000* encodes a cullin, which is a component of SCF ubiquitin ligase complexes involved in mediating responses to auxin, and the gene is implicated in more than 10 biological processes, including auxin-activated signaling pathway, cell cycle, ethylene-activated signaling pathway, and response to auxin. In general, these four candidate genes related to leaf should be further studied to gain an in-depth understanding of their mechanisms under shading.

**Table 4:** Annotation description of five gene groups based on GO analysis.

Group	Biological process	Glyma 1.1 ID	Annotation Description
I	phytohormone regulation	*Glyma06G293400*, *294600*, *295300*, *296200*, *296500*, *298500*, *299300*, *299900*, *300000*, *300100*, *300200*, *300300*, *300400*, *301000*, *301300*, *302300*, *303100*, *303600*, *304900; Glyma14G84700*, *86500*, *86600*, *87200*, *88200*, *88300*, *88400*, *88900; Glyma15G154000*, *154100*, *156100*, *157100*, *158300*, *159100*, *159200*, *161200*, *161300*, *161700*, *162600*, *163600*, *163700*, *166200*, *166800*, *167500*, *168100*, *168200*	abscisic acid-activated signaling pathway; auxin efflux/homeostasis/polar transport; auxin-activated signaling pathway; cellular response to ethylene stimulus; ethylene/gibberellin biosynthetic process; ethylene-activated signaling pathway; jasmonic acid and ethylene-dependent systemic resistance; negative regulation of abscisic acid-activated/ethylene-activated signaling pathway; response to abscisic acid/auxin/ethylene/gibberellin
II	photosynthesis	*Glyma06G293800*, *294600*, *295200*, *296500*, *297300*, *298300*, *299100*, *300900*, *301000*, *301100*, *301300*, *301500*, *302100*, *302200*, *302300*, *303600*, *305000; Glyma14G85300*, *85400*, *85600*, *85800*, *87200*, *87600*, *88300*, *88700*, *89000; Glyma15G154300*, *155600*,*156400*, *156600*, *156900*, *158900*, *159100*, *159200*, *159700*, *161600*, *161700*, *161800*, *161900*, *162000*, *162300*, *162500*, *163600*, *164700*, *167500*, *168100*	blue/far-red light signaling pathway; carbohydrate biosynthetic process/derivative biosynthetic process/metabolic process/transmembrane transport; cellular response to absence of light/light stimulus; photosystem II assembly/repair; photosynthesis, light reaction; response to absence of light/high light intensity/light stimulus/low light intensity stimulus/red or far red light/sucrose; starch catabolic/metabolic process
III	cellular processes	*Glyma06G293400*, *293800*, *293900*, *294100*, *294200*, *294400*, *294500*, *294600*, *295300*, *296000*, *296500*, *296800*, *299200*, *299700*, *299900*, *301000*, *301100*, *301500*, *301700*, *301900*, *302100*, *302300*, *302600*, *302700*, *303600*, *303700*, *304900; Glyma14G84700*, *84900*, *85100*, *85200*, *85400*, *85500*, *86100*, *86200*, *86500*, *86900*, *87100*, *87400*, *87500*, *87600*, *87700*, *87900*, *88300*, *88400*, *88600; Glyma15G154000*, *154100*, *154300*, *154600*, *154700*, *154900*, *155300*, *155400*, *155600*, *155700*, *156400*, *156500*, *157000*, *157100*, *157300*, *158300*, *158900*, *159100*, *159200*, *160900*, *161200*, *161300*, *161500*, *161600*, *161700*, *161800*, *161900*, *162000*, *162200*, *162500*, *163500*, *163700*, *163900*, *164100*, *164500*, *164600*, *164700*, *164900*, *165000*, *165200*, *166600*, *166700*, *166800*, *166900*, *167500*, *168100*	cell cycle/death/development/differentiation/division/growth/proliferation; cell wall organization/modification involved in abscission; cellular aromatic compound metabolic process/biosynthetic process/catabolic process/component organization/developmental process/macromolecule biosynthetic process/metabolic process/process involved in reproduction/response to stimulus; regulation of cellular process/biosynthetic process/component organization/macromolecule biosynthetic process/metabolic process; programmed cell death; unidimensional cell growth
IV	morphogenesis	*Glyma06G295300*, *296800*, *297400*, *300900; Glyma14G85400*, *88200*, *88900; Glyma15G154000*, *157100*, *161200*, *168100*	cell/leaf/simple leaf morphogenesis; cell morphogenesis involved in differentiation; cellular component morphogenesis; photomorphogenesis; regulation of photomorphogenesis
V	leaf development	*Glyma06G293800*, *295300*, *295700*, *296200*, *296500*, *296800*, *299200*, *299900*, *300300*, *301300; Glyma14G84700*, *85500*, *85600*, *85800*, *87600; Glyma15G154700*, *154800*, *155000*, *155300*, *156600*, *156900*, *157100*, *158300*, *159100*, *159200*, *161400*, *161500*, *161600*, *161800*, *162000*, *162600*, *168200*	leaf vascular tissue pattern formation/development/senescence; nitrogen compound metabolic process; response to water deprivation/nitrogen compound; regulation of response to water deprivation/nitrogen compound metabolic process/phosphorus metabolic process; water transport; cellular response to phosphate starvation; cellular nitrogen compound biosynthetic/metabolic process; organonitrogen compound metabolic process

### Candidate gene validation

**Figure 4:**
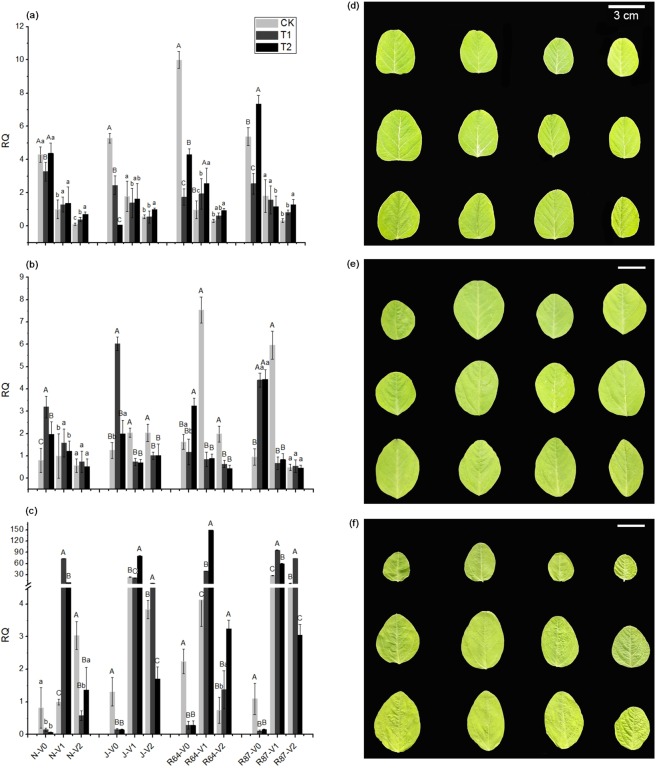
Comparison of the relative expression levels of *CCNA* (**a**), *CUL1* (**b**), and *GH3* (**c**) in ‘Nandou 12’ (N), ‘Jiuyuehuang’ (J), RIL64 (R64), and RIL87 (R87) under normal light (CK), one layer of shading net (T1), and two layers of shading nets (T2) at 7 days (V0), 15 days (V1), and 23 days (V2) after germination. ‘Nandou 12’, ‘Jiuyuehuang’, RIL64, and RIL87 (from left to right) in the photos were grown under CK, T1, and T2 (from bottom to top), whose fully developed unifoliate leaves, unrolling of first trifoliate leaves, and unrolling of second trifoliate leaves were sampled at 7 days (**d**), 15 days (**e**), and 23 days (**f**) after germination, respectively. The capital letters and the small letters on the top of SD bar denote significance level of *P* < 0.01 and 0.05 under different treatments, respectively.

qRT-PCR was performed on total RNA of leaves using gene-specific primers listed in Supplementary Table S3. According to the real-time PCR results, the expression profiles of each gene were shown (Fig. 4), and the expression level of *Gmact11* in CK ‘Nandou 12’ (N) at V1 was 1. *CCNA* was present at the highest level in CK ‘Jiuyuehuang’ (J) and RIL64 (R64) at V0, while in CK N and RIL87 (R87) was lower than T2. *CUL1* in N and J had a similar pattern to that of R64 and R87, and the expression of CK N and J had the lowest level at V0, so did the T1 and T2 R64 and R87 at V1 and V2. *GH3* differential expression between CK and T2 was significant in J and R64 at V1, and the same as those between CK and T1 in N and R87.

Supplementary Table S4 showed that the phenotypic values of LDW, SLW, TLA, and SLA of four materials in this section were similar to those in fields. N and R87 is more shade tolerant than J and R64. By analysis of variance, the differences of each material among CK, T1, and T2 treatments were significant (*P* < 0.05). The above results show that the correlation between three candidate genes and leaf-related traits can be preliminarily determined.

## Discussion

In terms of excellent advantages, RILs were selected to construct the soybean genetic linkage map in this study. With innovations, such as new molecular markers, high-throughput sequencing has become the preferred strategy to obtain SNP markers for constructing a high-density linkage map for QTL mapping. Zhang *et al*.^19^ obtained 5,785 SNP markers by using 110 RILs (F_8_) derived from ‘Luheidou 2’ and ‘Nanhuizao’ and constructed a soybean genetic map with a total map distance of 2,255.18 cM and an average marker interval distance of 0.39 cM. Huang *et al*.^20^ constructed an asparagus bean map, which consisted of 5,225 SNP markers, spanning a total distance of 1,850.81 cM, with an average distance of 0.35 cM. In comparison with those above maps, the map in the present study contained more families in the population, obtained more SNP markers, and required a larger average marker interval distance.

The results of QTL identification revealed that *qCLN17-3*, *qTLA12-41*, *qTLA15-99*, *qLDW12-40*, *qLDW15-98*, and *qLDWR5-19* were major QTLs. Three major QTLs have been detected in other studies: *qTLA15-99* and *qLDW15-98* (12,783,197–15,034,369 bp) were located in 9,396,584–34,757,105 bp on Chr15^8^, and *qLDWR5-19* (3,116,523–3,201,469 bp) is found in 209,284–3,735,646 bp on Chr05^12^. These QTLs are associated with the leaflet area.

Many QTLs related to 43 traits are located near the regions of the three QTL clusters. Chen *et al*.^21^ detected a QTL related to leaflet length (7,922,227–41,622,802 bp) was located near *qCLN14-100* and *qLDWR14-95* (7,440,291–8,071,592 bp) on Chr14, while Kim *et al*.^15^ identified two QTLs related to leaflet shape and width (13,755,345–17,021,739 bp) were located near *qTLA15-99* and *qLDW15-98* (12,793,197–15,034,369 bp) on Chr15. Reinprecht *et al*.^22^ and Keim *et al*.^23^ reported a QTL associated with seed weight (48,441,504–48,467,115 bp) and first flowering (48,305,238–48,441,504 bp), respectively. The two QTL regions were similar to *qTLA6-192* and *qLDW6-192*, which were detected on Chr06 in 2016 with an interval of 48,180,859–49,430,702 bp. In another study, a QTL associated with seed weight is located in 5,540,205–16,352,945 bp^24^, and a QTL related to sudden death syndrome is found in 3,063,657–9,434,565 bp^25^. Both of them were found on Chr14. Interestingly, *qCLN14-100* and *qLDWR14-95*, which respectively controlled the compound leaf number and the ratio of leaf dry weight in our study, were detected within the two regions. Moreover, *qTLA15-99* and *qLDW15-98* located on Chr15 were within the regions associated with shoot weight, nodule number^26^, pod maturity^27^, seed width^28^, sudden death syndrome^25^, and lodging^29^.

According to the results of gene expression, the correlation between leaf-related traits and three candidate genes can be found. In the case of normal light, *CCNA* expression level in shade-tolerance materials was higher. However, *CUL1* and *GH3* were highly expressed in all materials under shade, and these expression levels varied both with the shading degree and the shade-tolerance of materials. *Glyma06G296500* (*GH3*) was associated with the auxin responsive *GH3* gene family according to Phytozome v12.1 (https://phytozome.jgi.doe.gov/pz/portal.html). Wu *et al*.^30^ found there were some auxin response factors regulating tomato leaf shape development. In conclusion, the expression level of these three candidate genes in leaves was related to the shading condition and the shade-tolerance of materials, and the effect of *CUL1* and *GH3* on leaf-related traits may be contrary to that of *CCNA*.

## Methods

### Plant materials and experiment design

The RILs of 200 individuals were developed from a cross between the cultivar ‘Nandou 12’ (female) and the Sichuan local variety ‘Jiuyuehuang’ (male). ‘Nandou 12’ has lodging resistance and strong shade tolerance, which are significantly different from the phenotypic characters of ‘Jiuyuehuang’.

The field experiments of monoculture and relay intercropping were conducted in the Modern Agricultural Base of Renshou County, Sichuan Province, China (104°09′N, 30°00′E). In these experiments, 137 F_7_ RILs and 200 F_8_ RILs were grown together with two parents in a randomized block design with two replications on June 15, 2015 and 2016, respectively. In monoculture planting, each plot included 25 plants with 0.5 m between rows and 0.1 m between plants. In relay intercropping with a wide-narrow row cropping pattern (two maize rows with a 0.4 m inter-row distance and two soybean rows with a 0.5 m inter-row distance), adjacent rows of maize and soybean spaced 0.55 m, and 0.16 m and 0.1 m between plants of maize and soybean, respectively. The row length was 2.5 m. ‘Zhenghong 505’ (*Zea mays* L.) as the semi-compact maize was cultivated in narrow rows on April 1, 2015 and 2016, and harvested on August 5, 2015 and 2016. The soybean was cultivated in wide rows, and the other conditions were the same as those of the monoculture.

According to the field experiment phenotype data, two parents, RIL64 (weaker shade tolerance), and RIL87 (stronger shade tolerance) were analyzed to gene expression by real-time PCR. The above four accessions were sowed under normal light (CK, PPFD = 878.15 μmol/m^2^/s), one layer of black sun-shade net (T1, PPFD = 336.79 μmol/m^2^/s), and two layers of black sun-shade net (T2, PPFD = 251.54 μmol/m^2^/s) with three repeats.

### Measurement of leaf-related traits

The soybean samples in field were collected 40 days after germination. Three normal samples with two repeats of each parent and RIL individual were selected to determine their leaf-related phenotype data. The following standards were used to evaluate the six leaf-related traits: CLN; TLA, which use the hole puncher get the round piece (diameter = 1.2 cm), then divide the hole area by the round piece weight and multiply by the total leaf dry weight; SLA, which is the ratio of the area of one side of a small round leaf piece to its dry weight; SLW, which is the reciprocal of SLA; LDW; and LDWR, which is the ratio of LDW to the sum of LDW and stem petiole dry weight.

‘Nandou 12’, ‘Jiuyuehuang’, RIL64, and RIL87 for candidate gene analysis were sampled to measure TLA, SLA, LDW, and SLW. The fully developed unifoliate leaves, unrolling first trifoliate leaves, and unrolling second trifoliate leaves were collected in 7 days (V0), 15 days (V1), and 23 days (V2) after germination under three treatments with repeated three times, respectively.

### DNA extraction

The young leaves of 2 parents and 200 individuals of RILs were collected 20 days after germination. DNA was extracted as described by Doyle and Doyle^31^ with slight modifications. In brief, 3–5 g of fresh leaves were grinded and powdered in a mortar with liquid nitrogen and placed in a pre-cooled 2 mL Eppendorf tube (load 1/3), then mixed into 65 °C 600 μL of preheated CTAB and 20 μL of hydrophobic base ethanol, and incubated in a water bath at 65 °C for about 1 h. The preparation was gently shaken once every 10 min. Afterward, 400 μL of Tris-balance phenol and 400 μL of chloroform iso-amyl alcohol solution were added (24:1), fully mixed and gently shaken for 15 min. The samples were centrifuged for 10 min at 10,000 rpm. Then, 500 μL of the supernatant was transferred into a new Eppendorf tube, finished once suction filter with 400 μL of chloroform iso-amyl alcohol solution (24:1). The resulting mixture was centrifuged at 10,000 rpm for 10 min, and 400 μL of the supernatants was transferred into a new Eppendorf tube and added with 1 mL of pre-cooled isopropyl alcohol. The mixture was blended gently until the flocculent precipitated completely. The flocculent was gently selected from the mixture by using a syringe, transferred into a new Eppendorf tube, washed twice with 70% ethanol, then washed with anhydrous ethanol, blow dried in a clean bench, and dissolved in 200 μL of TE buffer. Finally, NanoVue was used to detect DNA quality, and agarose gel electrophoresis was conducted.

### Genotyping

The DNA fragments of each parent were randomly sheared into ~500 bp by ultrasonication, and the sequencing library was constructed by end repair, 3′-plus A, plus sequencing connector, purification, and PCR amplification. The library was sequenced through the HiSeq^TM^ 2500 system (Illunima, USA) sequencing platform after passing quality inspection. A high-density genetic linkage map for 200 RILs was constructed with a SLAF-seq technology. The SLAF marker was identified and genotyped in accordance with the procedures described by Sun *et al*.^32^. The original sequencing of SLAF-seq library was PE125bp. After the raw data were filtered, the reads in the *Glycine max* genome sequence were compared using the BWA software, and sequences mapped in the same position were defined as one SLAF locus. The alleles of each SLAF locus were then defined in accordance with parental reads with a sequence depth of >15X fold, and each genotype sequence contained 30% of the offspring information. Only SLAFs with two to four alleles were identified as polymorphic and considered potential markers. All polymorphism SLAF loci were genotyped with consistency in parental and offspring SNP loci. The marker codes of the polymorphic SLAFs were analyzed on the basis of the RILs. High-quality SLAF markers for the genetic mapping were filtered to construct the map.

### Genetic map construction

Genetic map construction was based on 200 F_7_ RILs and was completed by using HighMap software by Beijing Biomarker Technologies (www.biomarker.com.cn). First, the recombination rate and the modified logarithm of odd (LOD) scores between markers were calculated to infer the linkage phases in chromosomes, through the maximum likelihood method for genetic map construction, according to the map order to rectify and sequence circularly, and a high quality genetic map was obtained.

### QTL analysis and GO enrichment

QTL analysis was performed through the inclusive composite interval mapping (ICIM)^33^ of QTL IciMapping 4.1. An QTL was significant with the LOD score of 2.5. The nomenclature of McCouch *et al*.^34^ was used as a reference with slight modifications. The name of the QTLs in this study included four parts. For example, *q*, *CLN*, *17*, and *3* of *qCLN17-3* represented QTL, trait abbreviation, chromosome number, and QTL position in this chromosome, respectively. QTL mapping was drawn by MapDraw^35^. QTL validation was conducted with the software of QTL.gCIMapping.GUI in data of this study (https://cran.r-project.org/web/packages/QTL.gCIMapping.GUI/index.html)^36^.

The topGO software was used to conduct enrichment analysis of the related genes between the sample groups annotated into the GO database, and the hierarchical relationship of the nodes significantly enriched in the GO system was demonstrated visually in the form of directed acyclic graph.

### RNA extraction, cDNA synthesis and real-time PCR

The leaves for RNA extraction were sampled with liquid nitrogen frozen in 7 days, 15 days, and 23 days after germination. RNA extraction was conducted according to the Plant RNA Kit (Omega Bio-tek, Inc., USA) instruction. The cDNA synthesis and real-time PCR were conducted according to the RT reagent Kit (Takara Bio, Inc., Japan) and SYBR Premix Ex Taq II (Takara Bio, Inc., Japan) instructions, respectively. Primers were designed by Takara Bio and synthesized by Sangon Biotech (Shanghai, China). Real-time PCR conducted with QuantStudio^TM^ 6 Flex System and calculated the RQ value with 2^(−∆∆CT)^ in Real-Time PCR Software v1.3 (Thermo Fisher, USA).

### Data statistics

Microsoft Excel 2013 was used for data-calculated analysis and the gene expression profiles. IBM SPSS Statistics 22 was utilized to conduct the ANOVA and correlation analysis of the leaf-related traits. Origin Pro 8.0 was employed to draw the histogram.

## Supplementary information

Supplementary Table S1, Supplementary Table S2, Supplementary Table S3, Supplementary Table S4
