# Supplementary material for: Genetic map construction and QTL analysis of leaf-related traits in soybean under monoculture and relay intercropping
Source: Sci Rep. 2019 Feb 25;9:2716. doi: 10.1038/s41598-019-39110-8 (PMC6390081; doi:10.1038/s41598-019-39110-8)
Supplement: Supplementary file 1 — Supplementary Table S1, Supplementary Table S2, Supplementary Table S3, Supplementary Table S4 [file 41598_2019_39110_MOESM1_ESM.pdf]

Title: Genetic map construction and QTL analysis of leaf-related traits in soybean under monoculture and relay intercropping

Authors: Dai-Ling Liu, Si-Wei Chen, Xin-Chun Liu, Feng Yang, Wei-Guo Liu, Yue-Hui She, Jun-Bo Du, Chun-Yan Liu, Wen-Yu Yang & Xiao-Ling Wu

Supplementary Table S1. Phenotypic correlations for leaf-related traits under monoculture (M) and relay intercropping (RI). **Correlation is significant at the 0.01 level, *correlation is significant at the 0.05 level.

(a) Phenotypic correlations in 2015

Traits	CLN(M/RI)	TLA(M/RI)	SLA(M/RI)	SLW(M/RI)	LDW(M/RI)	LDWR(M/RI)
CLN(M/RI)	1					
TLA(M/RI)	0.527**/0.715**	1				
SLA(M/RI)	-0.095/0.215**	0.315**/0.236**	1			
SLW(M/RI)	0.121/-0.22**	-0.295**/-0.227**	-0.982**/-0.987**	1		
LDW(M/RI)	0.594**/0.694**	0.857**/0.91**	-0.197**/-0.048	0.215**/0.051	1	
LDWR(M/RI)	0.066/-0.536**	0.299**/-0.622**	0.098/-0.137	-0.09/0.146*	0.248**/-0.603**	1

(b) Phenotypic correlations in 2016

Traits	CLN(M/RI)	TLA(M/RI)	SLA(M/RI)	SLW(M/RI)	LDW(M/RI)	LDWR(M/RI)
CLN(M/RI)	1					
TLA(M/RI)	0.747**/0.706**	1				
SLA(M/RI)	-0.179*/-0.246**	0.112/-0.085	1			
SLW(M/RI)	0.06/0.086	-0.115/-0.127	-0.677**/-0.54**	1		
LDW(M/RI)	0.802**/0.729**	0.941**/0.953**	-0.201**/-0.353**	0.09/0.091	1	
LDWR(M/RI)	-0.138/0.145*	0.025/0.376**	-0.075/-0.192**	0.206**/0.092	0.028/0.401**	1

Title: Genetic map construction and QTL analysis of leaf-related traits in soybean under monoculture and relay intercropping

Authors: Dai-Ling Liu, Si-Wei Chen, Xin-Chun Liu, Feng Yang, Wei-Guo Liu, Yue-Hui She, Jun-Bo Du, Chun-Yan Liu, Wen-Yu Yang & Xiao-Ling Wu

Supplementary Table S2. The information of three candidates' annotations.

Glyma 1.1 ID	Physical position	Arabidopsis homologues	GO biological process	GO molecular function
Glyma06G296500	48,552,102–48,556,924	*AT4G03400*	response to auxin, response to light stimulus	ligase activity
Glyma14G087100	7,787,745–7,791,461	*AT1G47220*	cell cycle, cell division, mitotic cell cycle, positive regulation of cell cycle, positive regulation of cell proliferation, regulation of cell cycle, regulation of cyclin-dependent protein serine/threonine kinase activity, regulation of mitotic nuclear division	cyclin-dependent protein serine/threonine kinase regulator activity, protein kinase binding
Glyma15G154000	12,786,071–12,798,883	*AT4G02570*	SCF complex assembly, auxin-activated signaling pathway, cell cycle, embryo development ending in seed dormancy, ethylene-activated signaling pathway, jasmonic acid mediated signaling pathway, leaf development, phloem or xylem histogenesis, protein ubiquitination, regulation of circadian rhythm, response to auxin, response to jasmonic acid, ubiquitin-dependent protein catabolic process	protein binding, ubiquitin protein ligase binding

Title: Genetic map construction and QTL analysis of leaf-related traits in soybean under monoculture and relay intercropping

Authors: Dai-Ling Liu, Si-Wei Chen, Xin-Chun Liu, Feng Yang, Wei-Guo Liu, Yue-Hui She, Jun-Bo Du, Chun-Yan Liu, Wen-Yu Yang & Xiao-Ling Wu

Supplementary Table S3. Primers used for qRT-PCR.

Gene	Primer name	Sequence (5'– 3')
Actin	Gmact11-qF	ATCTTGACTGAGCGTGGTTATTCC
	Gmact11-qR	GCTGGTCCTGGCTGTCTCC
Glyma06G296500	GH3-F	ACTCCTTTACTCACTCAACAACCCA
	GH3-R	GAACCCTTCCACCTTCCCTTAT
Glyma14G087100	CCNA-F	TTGGGTGGTCCGACAGTGAA
	CCNA-R	GGAATACAACCGATGCAGCC
Glyma15G154000	CUL1-F	TGTGCGTATTATGAAGAGTCGGA
	CUL1-R	GCTTCTTAATTGCCTTGACATCC

Title: Genetic map construction and QTL analysis of leaf-related traits in soybean under monoculture and relay intercropping

Authors: Dai-Ling Liu, Si-Wei Chen, Xin-Chun Liu, Feng Yang, Wei-Guo Liu, Yue-Hui She, Jun-Bo Du, Chun-Yan Liu, Wen-Yu Yang & Xiao-Ling Wu

Supplementary Table S4. Phenotypic performance of LDW, SLW, TLA, and SLA under normal light (CK), one layer of black sun-shade net (T1), and two layers of black sun-shade net (T2) at 7 days (V0), 15 days (V1), and 23 days (V2) after germination. Nan, 'Nandou 12'; Jiu, 'Jiuyuehuang'.

Material	Treatment	LDW (g)			SLA (cm^2/g)			SLW (g/m^2)			TLA (cm^2)		
		V0	V1	V2	V0	V1	V2	V0	V1	V2	V0	V1	V2
	CK	0.02	0.04	0.06	330.31	847.80	701.19	31.85	17.20	21.02	8.16	33.38	38.81
Nan	T1	0.02	0.03	0.03	389.25	762.16	788.02	27.20	18.90	18.47	8.80	25.22	26.99
	T2	0.02	0.02	0.01	418.67	838.48	768.47	23.89	17.20	19.75	6.40	12.63	7.35
	CK	0.02	0.04	0.05	281.76	646.47	780.51	36.93	22.29	18.47	6.44	28.78	42.78
Jiu	T1	0.02	0.04	0.05	511.37	838.48	756.96	21.01	17.20	19.11	9.65	32.43	42.23
	T2	0.01	0.03	0.03	525.52	1036.20	968.91	19.03	14.01	15.29	7.86	26.10	25.87
	CK	0.02	0.04	0.05	291.83	811.57	780.51	34.28	17.83	18.47	6.96	33.39	38.94
RIL64	T1	0.01	0.03	0.04	538.29	701.19	811.57	18.58	21.02	17.83	8.01	18.09	29.72
	T2	0.01	0.02	0.01	445.31	730.05	753.60	23.00	19.75	19.11	5.53	11.06	9.72
	CK	0.01	0.03	0.05	314.90	788.02	646.47	34.50	18.47	22.29	4.50	24.93	28.93
RIL87	T1	0.02	0.03	0.05	392.32	709.27	847.80	26.10	20.38	17.20	5.89	23.46	38.99
	T2	0.01	0.02	0.01	457.54	824.25	948.59	22.56	17.83	15.29	5.87	14.52	11.44
